# Lung ultrasound in the critically ill (LUCI) and the lung point: a sign specific to pneumothorax which cannot be mimicked

**DOI:** 10.1186/s13054-015-1030-6

**Published:** 2015-09-08

**Authors:** German Moreno-Aguilar, Daniel Lichtenstein

**Affiliations:** Institution Anestesiar, Carrera 43A # 1 Sur - 100, Medellín, Colombia; Medical Intensive Care Unit, Hôpital Ambroise-Paré, Paris-West University, Nanterre, France

We congratulate Drs Zhang and Chen for their interest in lung ultrasound [1]. They describe a sign mimicking a lung point. Would this mean that the lung point is not specific to pneumothorax?

First, in a standard approach to a suspected case of pneumothorax (in a routine examination or in cardiac arrest), the lung point must not be just found, but actively sought for. A “lung point” found by chance, especially anteriorly near the sternum, has little chance to be a pneumothorax—or means a very small one. Practically, the lung point must be sought for when, *and only when*, the first scan has shown an A’-profile of the BLUE-protocol: this means, at the anterior chest wall in supine patients, the association of abolished lung sliding, plus the A-line sign (see video A’-profile at [2]). When, *and only when*, an A’-profile has been detected should the operator search for a lung point, first laterally, then, if needed, posteriorly, and then near to the rachis (Fig. 1).

**Fig. 1:**
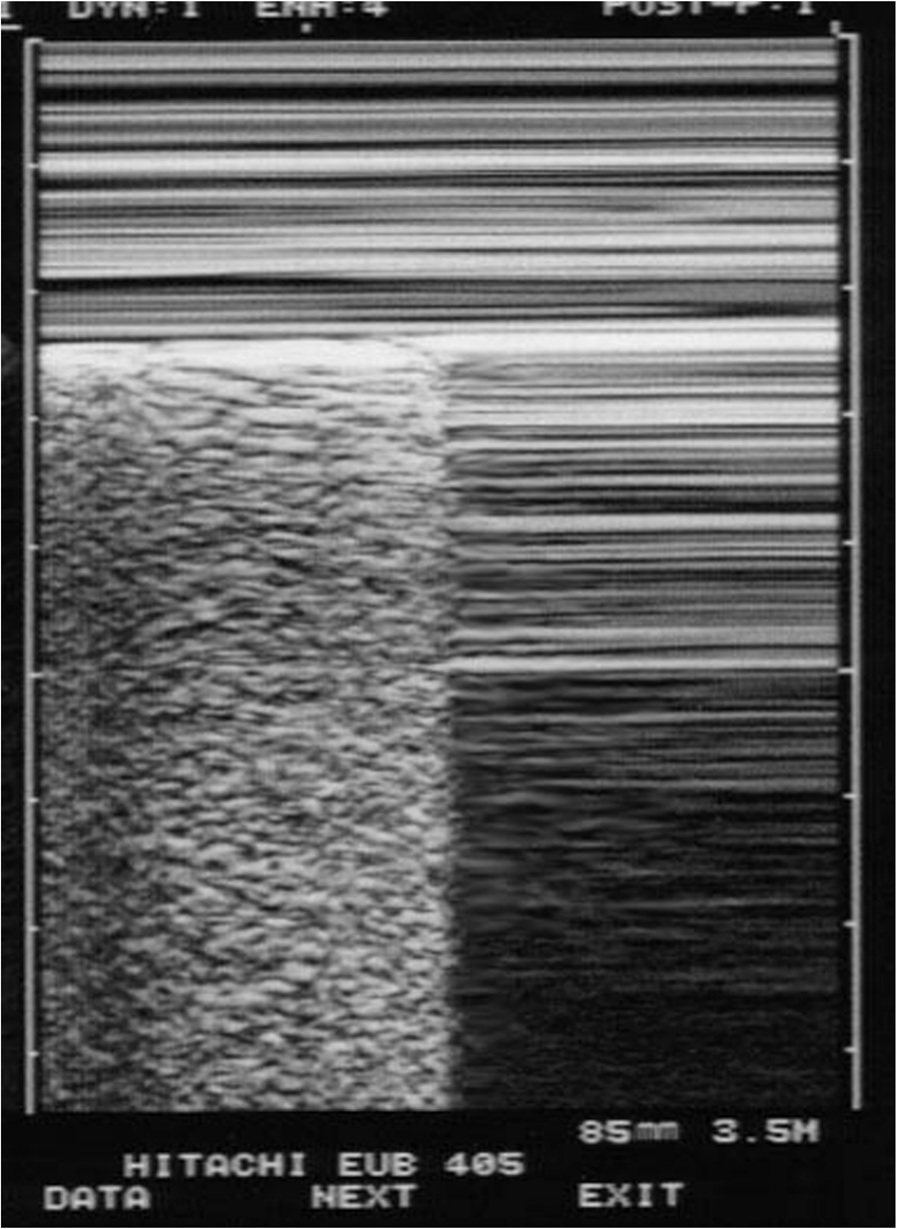
Regular M-mode outlook of a lung point. To the *left*, a seashore can be described, arising precisely from the pleural line. To the *right*, suddenly, a stratosphere sign is visible, that is, the total absence of any dynamic arising from the pleural line. An all-or-nothing rule, the lung point rules in pneumothorax

Second, looking at the data available in [1], we do not see any lung point. We see a change in the pattern of lung sliding: the seashore sign is modified but conserved, and is not replaced by a stratospheric pattern as it would be in the case of pneumothorax, generating the A’-profile. This change is sometimes seen and we did not allocate a specific name to it since it did not create any confusion with regard to our definition. We remind Drs Zhang and Chen that the lung point is defined as the alternation between the extended (lateral, posterior) A’-profile and any lung ultrasound sign: lung sliding, B-line, lung rockets (see video A’-profile at [2]). A fleeting vision of the heart (heart point) or a pleural effusion (swirl sign) is an equivalent. The confusion seen in [1] is not infrequent in the literature: some authors describe a false lung point in a lateral view near the diaphragm when the liver comes into the field of view on expiration [3]. The view of a living lung (lung sliding) replaced by a liver cannot be interpreted as representing a lung point: the liver shows an anatomic structure, and, as specified above, the A’-profile should first be identified; that is, anteriorly by definition in supine patients.

If used appropriately as described, lung ultrasound in the critically ill (LUCI) is a solid, standardized field of study whose limitations can be scientifically analyzed [4]. We reiterate our message: the simpler the unit and probe, the more accessible is LUCI.

## Abbreviation

LUCI: Lung ultrasound in the critically ill
